# Predicting the Impact of Climate Change on the Distribution of *Rhipicephalus sanguineus* in the Americas

**DOI:** 10.3390/su15054557

**Published:** 2023-03-03

**Authors:** Marcos Sánchez Pérez, Teresa Patricia Feria Arroyo, Crystian Sadiel Venegas Barrera, Carolina Sosa-Gutiérrez, Javier Torres, Katherine A. Brown, Guadalupe Gordillo Pérez

**Affiliations:** 1 Unidad de Investigación Médica de Enfermedades Infecciosas y Parasitarias, Centro Médico Nacional Siglo XXI, IMSS, Mexico City 06720, Mexico; 2 Department of Biology, University of Texas Rio Grande Valley, 1201 W University Drive, Edinburg, TX 78539, USA; 3 Instituto Tecnológico de Ciudad Victoria, Tecnológico Nacional de México, Blvd. Emilio Portes Gil 1301, Ciudad Victoria 87010, Mexico; 4 Instituto de Ciencias Agropecuarias, Universidad Autónoma del Estado de Hidalgo, Tulancingo 43600, Mexico; 5 The Oden Institute for Computational Engineering and Sciences, The University of Texas, Austin, TX 78712, USA; 6 Cavendish Laboratory, University of Cambridge, Cambridge CB3 0HE, UK

**Keywords:** species distribution model, ticks, climate change, MaxEnt, *Rhipicephalus sanguineus*

## Abstract

Climate change may influence the incidence of infectious diseases including those transmitted by ticks. *Rhipicephalus sanguineus* complex has a worldwide distribution and transmits Rickettsial infections that could cause high mortality rates if untreated. We assessed the potential effects of climate change on the distribution of *R. sanguineus* in the Americas in 2050 and 2070 using the general circulation model CanESM5 and two shared socioeconomic pathways (SSPs), SSP2-4.5 (moderate emissions) and SSP2-8.5 (high emissions). A total of 355 occurrence points of *R. sanguineus* and eight uncorrelated bioclimatic variables were entered into a maximum entropy algorithm (MaxEnt) to produce 50 replicates per scenario. The area under the curve (AUC) value for the consensus model (>0.90) and the partial ROC value (>1.28) indicated a high predictive capacity. The models showed that the geographic regions currently suitable for *R. sanguineus* will remain stable in the future, but also predicted increases in habitat suitability in the Western U.S., Venezuela, Brazil and Bolivia. Scenario 4.5 showed an increase in habitat suitability for *R. sanguineus* in tropical and subtropical regions in both 2050 and 2070. Habitat suitability is predicted to remain constant in moist broadleaf forests and deserts but is predicted to decrease in flooded grasslands and savannas. Using the high emissions SSP5-8.5 scenario, habitat suitability in tropical and subtropical coniferous forests and temperate grasslands, savannas, and shrublands was predicted to be constant in 2050. In 2070, however, habitat suitability was predicted to decrease in tropical and subtropical moist broadleaf forests and increase in tropical and subtropical dry broadleaf forests. Our findings suggest that the current and potential future geographic distributions can be used in evidence-based strategies in the design of control plans aimed at reducing the risk of exposure to zoonotic diseases transmitted by *R. sanguineus*.

## 1 Introduction

Global warming affects the geographic distribution of vectors transmitting infections between hosts, causing vector-borne diseases (VBDs). The World Health Organization estimates that VBDs are responsible for about 92 million human infections annually [1]. Ticks are among the most important pathogen transmission vectors, affecting animal and human health worldwide and causing significant economic losses [2]. There is increasing evidence to suggest that the incidence of tick-borne diseases (TBDs) is due to climate change [3], and that the spread of ticks into new geographical areas impacts the prevalence of infection transmitted by ticks in these areas [4]. Changing temperatures have a direct impact on the physiology of ticks. Increased temperatures generate faster development and a shorter life cycle, while reduced temperatures and high environmental humidity increase activity and reduce the mortality rates of ticks [5]. Several studies suggest that pathogens transmitted by *Rhipicephalus sanguineus* (*Ixodidae*, also known as the brown dog tick, kennel tick, or pantropical dog tick) [6] could emerge in new geographic regions in the future because of climate change [7].

Over 90% of bacterial species transmitted by ticks belong to two orders, *Spirochaetales* and *Rickettsiales* [8], both of which cause a broad spectrum of TBDs including Borreliosis, Rickettsiosis Anaplasmosis, and Ehrlichiosis, in humans and animals. TBDs are distributed globally in subtropical and tropical regions of the world including Central America and the Caribbean (CAC) [9]. *R. sanguineus* probably evolved as an ectoparasite of carnivores in tropical climates and with the domestication of the dog. This tick can feed on both humans and canines in a variety of environments [6]. *R. sanguineus* is the most widespread species of tick. It is typically found in tropical and subtropical regions and human activities are thought to greatly influence its spread in these regions [10]. Optimum environmental conditions for survival of this tick are temperature between 20 °C and 38 °C with a relative humidity between 35 to 95% [7], though it can also survive in arid conditions [11].

Despite the importance of R. sanguineus in public health and veterinary medicine, there is still a lack of information regarding the potential distribution of this species in geographical regions where recent cases of Rocky Mountain Spotted Fever (RMSF) have been reported such as Mexico and South America [12,13]. We hypothesize that climate change may be influencing the spread of R. sanguineus to these areas and elsewhere in the Americas. To address this gap in knowledge we have generated species distribution models (SDMs) of R. sanguineus and applied the CanESM5 (Canadian Earth System Model version 5) general circulation model (GCM) model to predict its potential suitable habitat in 2050 and 2070 using two shared socioeconomic pathways (SSPs), SSP2-4.5 and SSP5-8.5. The SSP2-4.5 represents a moderate emissions scenario while SSP5-8.5 represents a high emissions scenario [14–16]. Identifying new areas where ticks could potentially live may help to establish new preventive/control programs for TBDs, particularly in geographic areas where tick-borne diseases are currently emerging.

## 2 Materials and Methods

### 2.1 Study Area

The potential distribution of *R. sanguineus* was predicted in the Americas. Areas that experience annual temperatures that range from −7 to 29 °C (temperate regions) or 7 to 39 °C (tropical regions) and rainfall during 35–85% of the year are generally permissive environmental conditions for the presence of *R. sanguineus*. The accessibility area for the species or “M” (movement or dispersal over relevant periods) was delimited based on the map of ecoregions [17].

Geospatial locations of ticks were obtained from several sources. Fifteen percent of these data were from a database assembled from records from 18 states in Mexico over the past 20 years in the Medical Research Unit of Infectious Diseases, Instituto Mexicano del Seguro Social arthropod collection (prepared by GGP). Records of tick observations from the rest of the Americas were obtained from publicly available sources: the Global Biodiversity Information Facility; GBIF (https://www.gbif.org/10.15468/dl.tz7xbz, accessed on 15 August 2022) (76% of the data) [18]; and observations published in scientific papers (9% of the data). SDMs were generated using historical climate data to predict the current (1970–2021) and future (2041–2070) potential species distribution of *R. sanguineus* using CanESM5 (version 5) [15]. Climatic variables not correlated with temperature and precipitation conditions were included from areas where *R. sanguineus* observations were recorded (Table 1).

### 2.2 Presence Records

**Figure 1:**
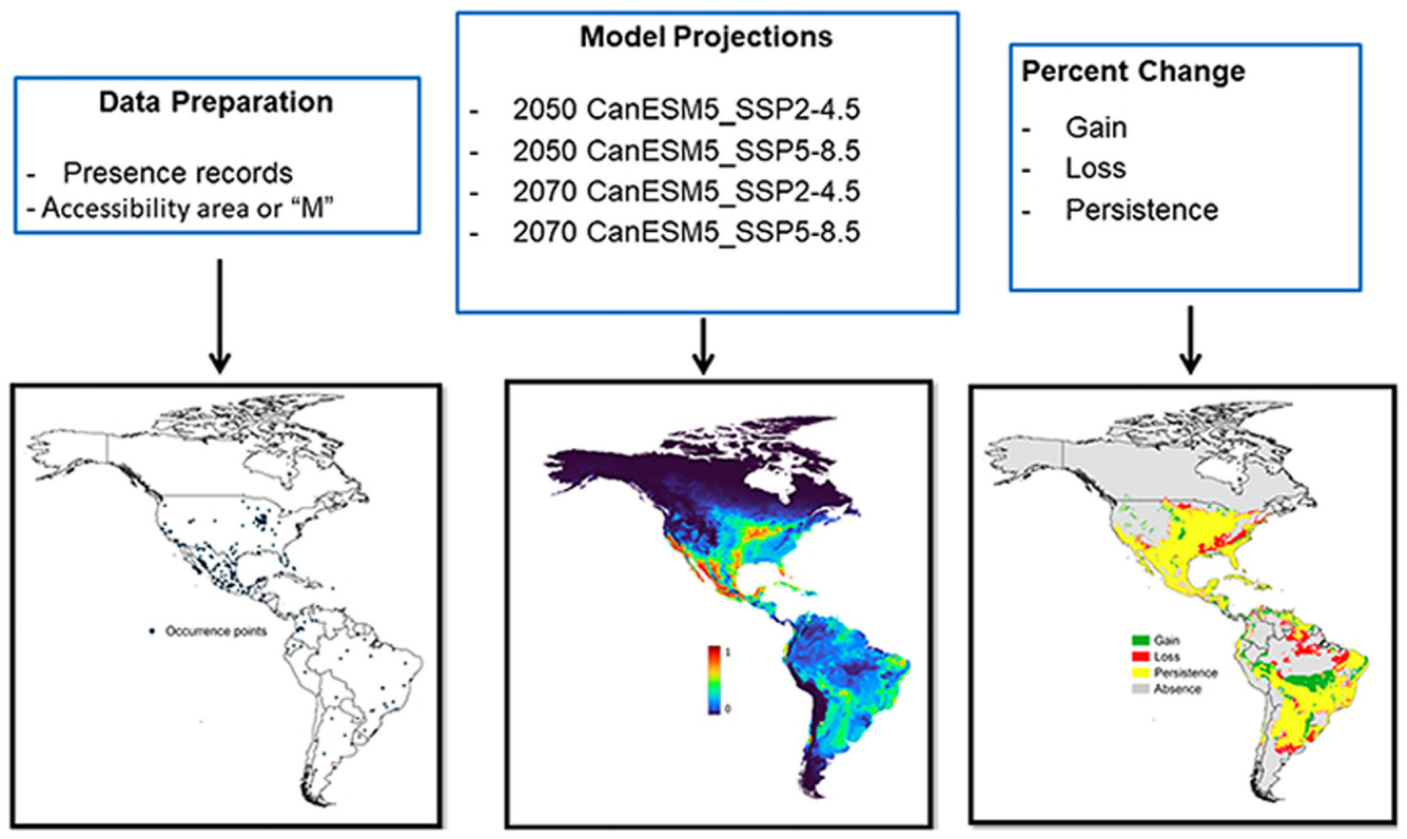
Processes used to generate SDMs for *R. sanguineus*.

The assembled database contained 7715 records of *R. sanguineus*. To clean the data, we only retained records that were obtained from a single occurrence. We removed duplicated records from multiple sources, records lacking geographic information or with the geographic coordinate “(0,0)”, as well as records from the oceans and seas. Through this process, a total of 355 records were obtained representing unique geographic locations where *R. sanguineus* was observed and SDMs (Figure 1) were generated using a random sampling of 70% from the internal calibration dataset as training data. The remaining 30% formed the validation dataset. First, internal calibration and internal validation datasets were used in consensus preselection algorithms. The internal calibration dataset was then used to calibrate individual models before using it for internal validation [18].

### 2.3 Bioclimatic Predictor Variables

Bioclimatic variables with potential biological relevance for the distribution of *R. sanguineus* were selected based on the average daytime temperature ranges, 20–30 °C from spring to autumn [19]. For current conditions, a set of 19 bioclimatic variables (www.worldclime.org, accessed on 9 September 2022) were used with a spatial resolution of 30 arc seconds (≈1 km^2^) (interpolations of observed data from 1960–1990) [18]. To avoid multicollinearity of these variables, highly correlated bioclimatic variables were removed with Pearson correlation > 0.9 and with a variance inflation factor (VIF) value of 5 [20]. In addition, principal component analysis (PCA) was carried out to identify those variables with similar variations. The first two components extract 73.5% of variations of bioclimatic variables. Bioclimatic variables with similar variations were near to canonical spaces. Results from VIF analyses and PCA were combined to identify bioclimatic variable relationships. The bioclimatic variables 04 and 07 were removed from predictions due to the presence of a negative relationship with bioclimatic variable 13.

The variables retained were used to construct the models were: mean diurnal range (BIO2), isothermality (BIO3), mean temperature of warmest quarter (BIO10), precipitation of wettest month (BIO13), precipitation of driest month (BIO14), precipitation seasonality (BIO15), precipitation of warmest quarter (BIO18) and precipitation of coldest quarter (BIO19) (Table 1) [18]. These eight bioclimatic variables were downloaded using the GCM CanESM5 and two shared socioeconomic pathways (SSP) for scenarios SSP 2-4.5 and SSP 5-8.5 [16]. SSP2-4.5 represents a moderate emissions scenario in which temperatures are forecast to rise by about 1.5 °C by the end of the 21st century. SSP5-8.5 represents a high emissions scenario with an expected increase in temperature over 2 °C [16].

### 2.4 Model Construction

**Figure 2:**
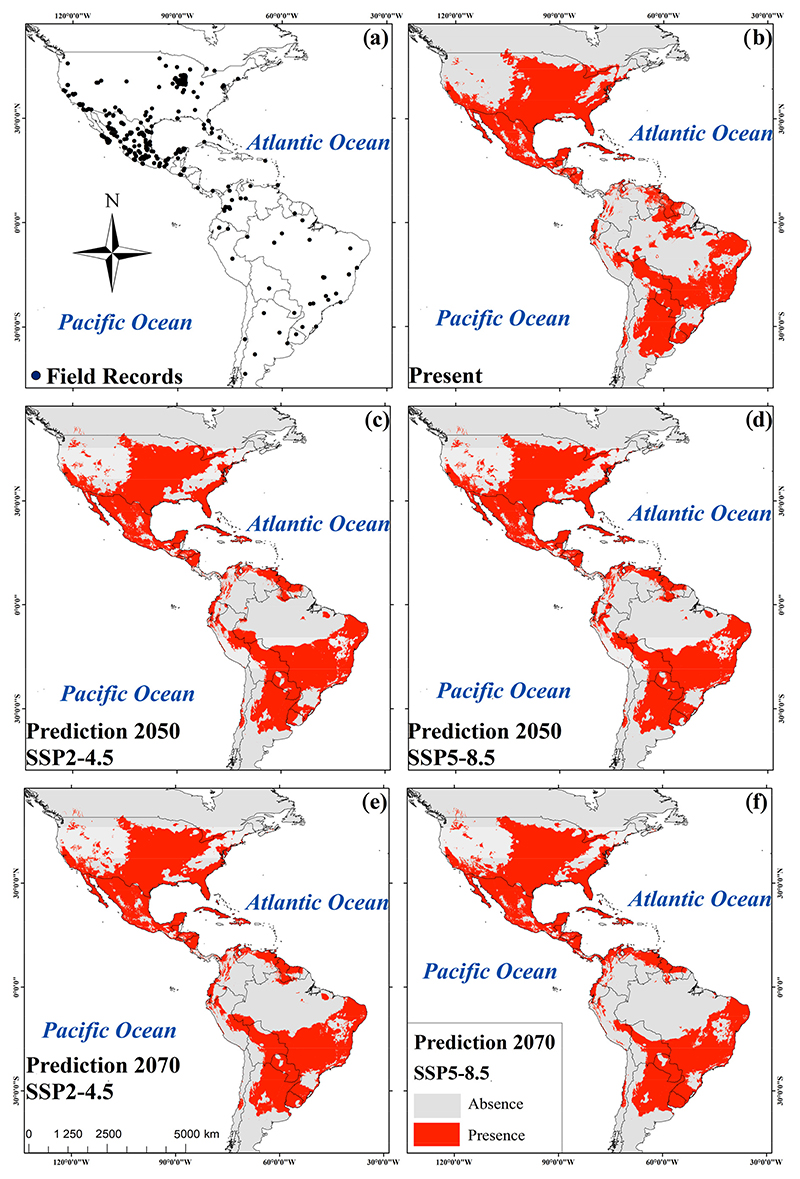
Field records of *R. sanguineus* in America (**a**); consensus maps of *R. sanguineus* for present (**b**); prediction to 2050 under the SSP2-4.5 scenario (**c**); prediction to 2050 under the SSP5-8.5 scenario (**d**); prediction to 2070 under the SSP2-4.5 scenario (**e**); and prediction to 2070 under the SSP5-8.5 scenario (**f**).

**Figure 3:**
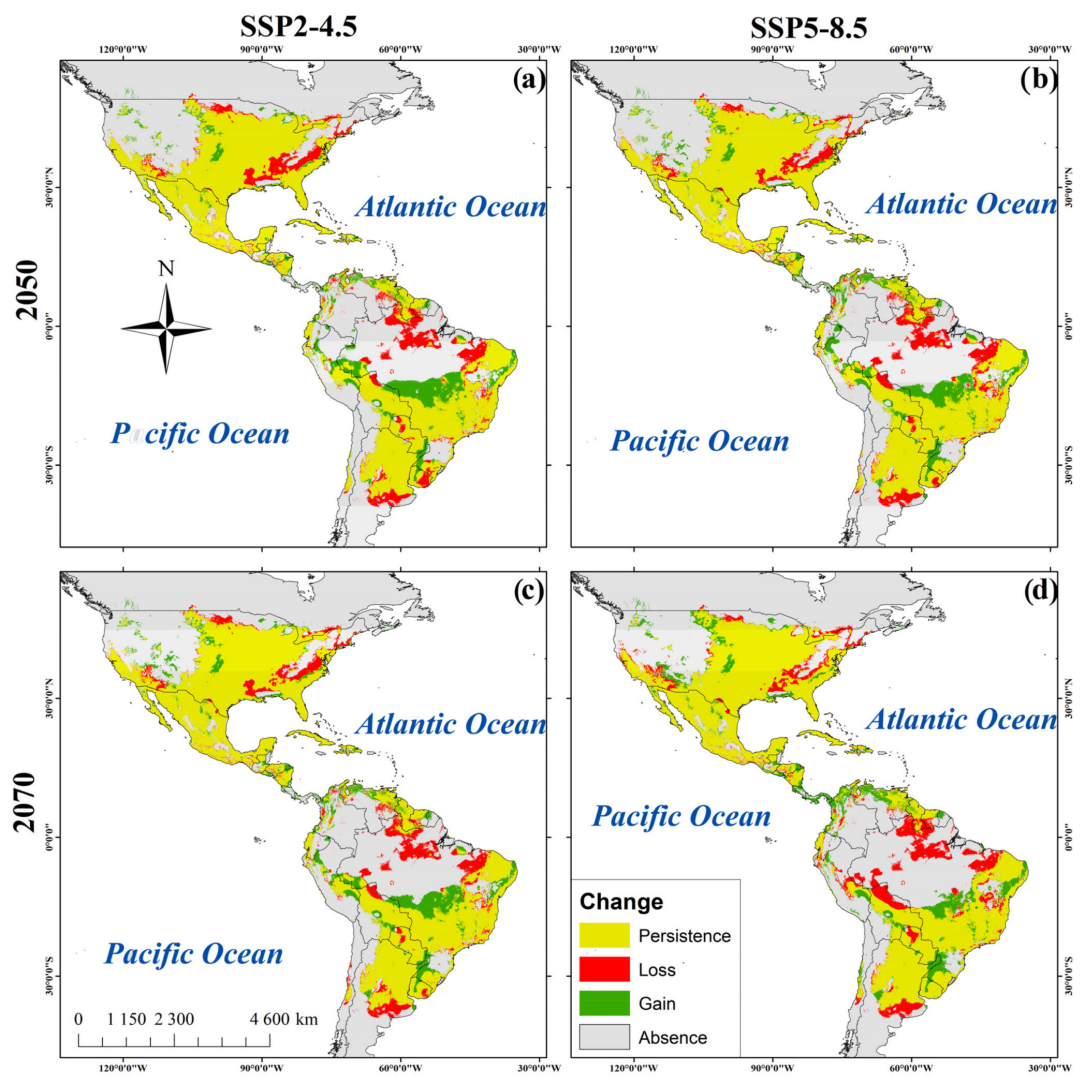
Habitat suitability maps of *R. sanguineus*. (**a**). SDM of *R. sanguineus* in the 2050_SSP2-4.5. (**b**) 2050 _SPP5-8.5. (**c**) 2070_SSP2-4.5. (**d**) 2070_SSP5-8.5.

Potential current and future geographic distributions *R. sanguineus* were predicted using a Kuenm R package for detailed calibration and construction of SDMs using a maximum entropy approach (MaxEnt) [21]. The models were generated by using default parameters and the bootstrap function in MaxEnt. Fifty replicates were run for current environmental conditions as well as for each of the two models of general circulation at 4.5 and 8.5 SSP for the years 2050 and 2070 [22]. The area under the curve (AUC) was estimated using a receiver operating characteristic (ROC) plot to evaluate model performance [18]. Consensus models were produced from the 50 replicates in an effort to reduce the predictive uncertainty of single models [18]. In total, five consensus maps were generated: one for present environmental conditions, two for 2050, and one for the year 2070, each with their respective SSP2-4.5 and SSP5-8.5 scenarios (Figure 2). Consensus models were converted to binary models using the “Fixed Cumulative Value 10” threshold acquired from MaxEnt. This low threshold value produced a wider distribution of *R. sanguineus* with an omission error close to zero [18]. The binary map corresponding to present environmental conditions was combined with the binary predictions for 2050 and 2070 scenarios SPP2-4.5 and SPP5-8.5 (Figure 3). The resulting maps were classified into four categories: presence, absence, increase, and reduction of habitable areas for the tick [21].

### 2.5 Model Evaluation

AUC values generated by Maxent and the partial ROC (pROC) test [18] were calculated for each of the replicates and the averaged final map. Values ranging from 0.9 to 1 imply that the model has remarkable predictive accuracy, while values ranging from 0.8 to 0.9 have good accuracy, and between 0.7 and 0.8 have regular performance. AUC values below 0.7 indicate poor and/or failed predictive ability and the resulting outputs were not considered [18]. The pROC test was calculated using Kuenm. A ratio > 1 indicated that the model had performed better than by random chance [21].

### 2.6 Final Model

**Figure 4:**
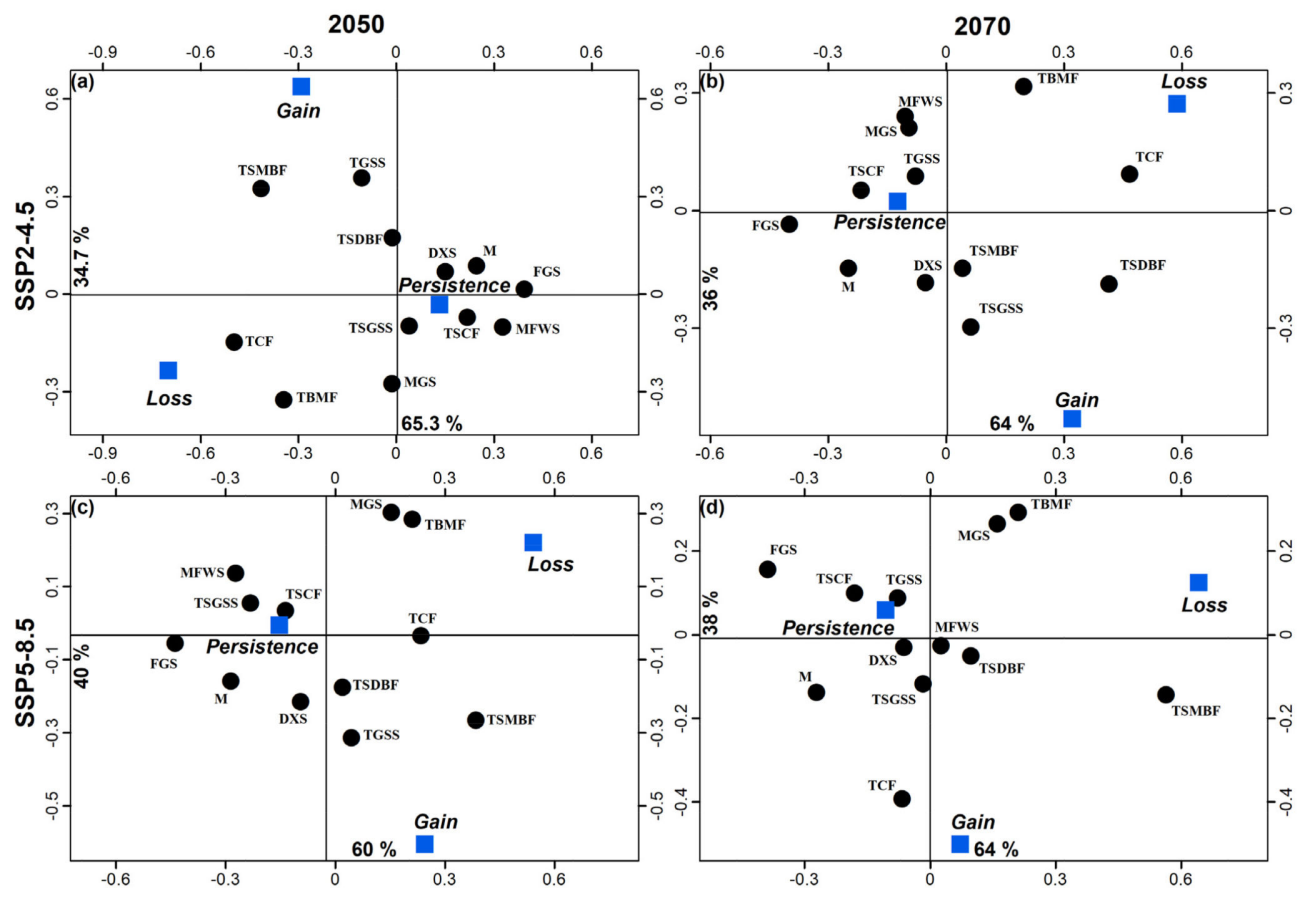
Correspondence analyses that associate changes in geographic distribution (persistent presence, loss, and gain) with 12 biomes using the 2050 and 2070 SDMs generated for the SSP2-4.5 and SSP5-8.5 scenarios. DXS = deserts and xeric shrublands, FGS = flooded grasslands and savannas, M = mangroves, MFWS = Mediterranean forest woodlands and scrub, MGS = montage grasslands and shrublands, TBMF = temperate broadleaf and mixed forests, TCF = temperate conifer forests, TGSS = temperate grasslands, savannas, and shrublands, TSCF = tropical and subtropical coniferous forests, TSGSS = tropical and subtropical grasslands, savannas and shrublands, and TSMBF = tropical and subtropical moist broadleaf forests.

The potential future distribution was generated for 2050 and 2070. Here, model performance is assessed based on statistical significance (pROC) and omission rates (OR), and Akaike’s information criterion is corrected for small sample sizes (AICc). For the pROC analysis of the generated models, 50% of the records were used. A reliability of 95% was obtained using 50 replicates through a bootstrap resampling and setting an omission error of 5%. The test generates values from 1 to 2, where a value with an average radius of 1 represents a model generated randomly [18]. We selected the model with delta AICc ≤ 2 from those that were statistically significant and had omission rates below 5%. The AICc is a model selection criterion that outperforms other available criteria (for example, AUC) for comparisons of different models generated using MaxEnt, particularly for small sample sizes [21]. AUC and pROC values of the final distribution consensus model were produced from eight low-correlation bioclimatic variables and 355 spatially rarefied occurrences. The test value of each AUC was multiplied in the QGIS (Geographic Information System) calculator by adding each value and then dividing by 50 (https://www.qgis.org/es/site/, accessed on 11 July 2022) [22]. The pROC values ranged from 1.2829 to 1.3856 (Table 2) indicating that models obtained were better than random predictions and that the predictions aligned well with areas of known occurrences of *R. sanguineus* (Figure 4).

### 2.7 Calculating Percent Change of Geographic Distribution and Model Evaluation

Correspondence analysis was used to associate changes in distribution (persistence presence, loss, and gain) with 12 in the Americas biomes (Figure 4). The percentage of change distribution between the current distribution model and each respective future climate change binary model was used to identify areas in the Americas with loss or gain of suitable habitat. The biomes layer was obtained from Dinerstein [17]. The association of biomes in these analyses was generated using a simple correspondence analysis in km^2^. Correspondence analysis is a multivariate ordination technique modified from the *X*^2^ test, which uses a contingency table to associate the classes of two categorical variables and creates a Cartesian diagram based on the association between the classes [23]. The objective of using correspondence analysis was to create a graph with the relative position of the categories of the studied qualitative variables. The positions of the categories of the variables reflected the degree of association between them.

## 3 Results

Areas of high suitability were found mainly throughout the U.S. and Mexico extending towards parts of Central and South America (Figure 2b–f). The association of biomes with predicted changes between current environmental conditions, 2050 and 2070 was significant under the SSP scenario 4.5 (*X*^2^_gl=22_ = 144.7, *p* < 0.001 and *X*^2^_gl=22_ = 115.4, *p* < 0.001, respectively). For both SSPs (4.5 and 8.5), the greatest loss of species was predicted to occur in flooded grasslands and savannas (FGS) and temperate conifer forests (TCF). An increase in potential species distribution was predicted in tropical and subtropical moist broadleaf forests (TSMBF) and tropical and subtropical grasslands, savannas, and shrublands (TSGSS). Biomes where geographical distributions were predicted to be constant are deserts and xeric shrublands (DXS), temperate broadleaf and mixed forests (TBMF), Mediterranean forests, woodlands, and scrub (MFWS), and tropical and subtropical coniferous forests (TSCF) (Figure 4). The association of biomes with predicted changes between the current environmental conditions, 2050 and 2070 was also significant under scenario 8.5 (*X*^2^_gl=22_ = 115.4, *p* < 0.001 and *X*^2^_gl=22_ = 108.0, *p* < 0.001, respectively). In 2050 the greatest loss of suitable habitat was predicted to occur in FGS, while an increase was predicted to occur in the TSMBF. The biomes in which the geographic distribution of *R. sanguineus* was predicted to remain constant were TCF, DXS, TBMF, MFWS, and TSCF (Figure 4). In contrast, in 2070 the greatest loss of suitable habitat was predicted to occur in TSMBF, while an increase was predicted in tropical and subtropical dry broadleaf forests (Figure 4).

For 2050, SSP2-4.5 predicted a 5.3% increase in suitable habitable areas while SSP2-8.5 predicted 5.36% (Table 3). Areas of increased habitat suitability included western and midwestern regions of the U.S., Panama, northern Peru and Venezuela, eastern Colombia and northeast and midwestern regions of Brazil. Reductions of potential distribution were predicted in regions of Paraguay, northern Bolivia, and Argentina (Figure 3a,b) (Table 3). However, for the year 2070 the SSP2-4.5 emissions scenario predicts a reduction of 5.2% of habitat suitability and this figure increases to 6.2% using SSP-8.5. The reductions occurred in regions of southern Ontario, Canada, in the U.S. states of Colorado, Arizona, Alabama, and Tennessee, in Coahuila, Mexico, in the southern Venezuela and Guyana, and in northern Brazil, Bolivia, and eastern Argentina. Thirty percent of the present area of habitat suitability remains unchanged in the 2050 and 2070 predictions (Table 3 and Figure 3a–d).

## 4 Discussion

Global warming affects the geographic distribution of vectors, increasing the incidence of the infectious diseases they transmit, and causing significant economic losses in livestock and human health [1]. In the last 20 years, the geographical distribution of *R. sanguineus* has increased notoriously and so has the incidence of RMSF reported by the CDC and other agencies [13]. Still, climate change models for the projection of *R. sanguineus* distribution are scarce. The 2–3 °C increase in average temperatures during the summer months (April to September) could favor the establishment of tick populations in previously tick-free areas, including temperate regions [7].

We forecast, for the first time, the potential distribution and possible expansion of *R. sanguineus* in areas of the Americas that had not been previously anticipated, such as the western and northern regions of the U.S., the northern part of Mexico, and previously unreported regions of Central and South America. In addition, our study shows that habitable areas for *R. sanguineus* would remain unchanged in temperate regions of southern Canada, the northern and northwestern regions of the U.S., and Argentina in South America under the 4.5 scenario for both the years 2050 and 2070.

Alkishe et al. presented a global distribution of *R. sanguineus* from five regions of the world using 368 data points and reported that this species is distributed differently between tropical and temperate zones [24]. Our study of the Americas showed an increase in habitat suitability for *R. sanguineus* in tropical and subtropical moist broadleaf forests. Habitat suitability was also shown to remain constant in TSCF, TCF, TBMF, MFWS, as well as in DXS. Loss of suitability was observed in FGS. In 2050 using the high emissions scenario of 8.5, habitat suitability remained constant in TSCF and TGSS. In 2070 there is a predicted loss of habitat in TSMBF, as well as an increase in suitable habitat in tropical and subtropical dry broadleaf forests. Previous studies in North America predicted areas of species expansion for *R. sanguineus* in Canada, and a study in Mexico reported that in addition to bioclimatic variables, the type of soil, vegetation, and annual precipitation were also strongly associated with the distribution of this species [25]. In this study, we found that the suitable habitat for *R. sanguineus* could increase in the western US, in Central America, and in some South American countries.

Previous studies on the potential distribution of *R. sanguineus* were limited to Mexico [25], the U.S., and Canada [24]; however, our results show a potential shift of this species’ distribution expanding to new regions. *R. sanguineus* is the most important vector for *Rickettsia rickettsii* in humans, which causes RMSF. The incidence of this disease has increased over the last two decades in the U.S. and Latin America [13]. In 2005, outbreaks of RMSF were reported in the eastern part of Arizona and on the U.S.-Mexico border. The common brown dog tick (*R. sanguineus*) was implicated as a vector of *R. rickettsii* [26] owing to the high prevalence of the disease in dogs [27]. In recent decades, cases of *R. rickettsii* with high lethality have been reported in the Baja California and Sonora states of Mexico [28,29]. Our study suggests that RMSF cases will continue to increase due to the increase in suitable habitable areas for *R. sanguineus*.

The annual number of days over 38 °C (100 °F) is expected to markedly increase in the next decade, leading to an increasing concern for the heat-driven emergence of tick-borne disease [30]. The seasonality of *R. sanguineus* has been studied in different parts of the world [12,24,31] and these studies found that temperature is the most important factor driving the population dynamics of this tick. Additionally, there is evidence that *R. sanguineus* can be more aggressive to humans at high ambient temperatures [8], and attachment and initiation of feeding also occurs more rapidly at high temperatures [11]. The number of eggs a tick produces is directly correlated with temperature. During the fall and winter, female oviposition requires a minimum threshold temperature (around 10 °C), and low temperatures decrease the survivability of this tick [32]. Thus, the risk of disease transmission to humans may increase during periods of warm weather and events associated with climate change have the potential to result in more frequent Rickettsiosis outbreaks [11]. In tropical areas of the Americas where temperatures are warm, the areas that are endemic for the presence of *R. sanguineus* have increased [19]. Temperatures from 30 °C to 35 °C in spring and summer favor the lifecycle of *R. sanguineus*. However, where temperatures reach from 37 °C to 42 °C in the summer, the tick cannot survive. In Mexico, the seasonality of precipitation is constant in tropical zones and variable in temperate climates [19]. *R. sanguineus* is thus more localized in coastal areas of Mexico where its biological cycle is more constant. This study predicts areas of potential distribution of *R. sanguineus* on the coasts between the Tropics of Cancer and Capricorn, where temperatures are optimal for the maintenance of the tick’s biological cycle. This study also predicts that suitable habitat can extend to new potential areas in the eastern U.S., southern Colombia, northern Peru, and southern and eastern Brazil as a result of climatic change.

Previous studies using calibration models showed stable areas of habitat for *R. sanguineus* in the eastern United States, the northern and southern states of Mexico, northern South America and Brazil, Europe, northern Africa, sub-Saharan African countries, Asia, and Australia [24]. In this study, we show an increase in suitable habitat in 2050 and 2070 in the eastern U.S., southern Colombia, northern Peru, and southern and eastern Brazil.

Reports from 20 years ago placed *R. sanguineus* between latitudes 50° N and 30° S [33,34]. The tropical lineage is proposed to be distributed between the Tropics of Cancer in the North and the Tropic of Capricorn, while the temperate lineage is found at the extremes of each of the South American tropics and below the Tropic of Capricorn where the Southern part of Brazil, Paraguay, Uruguay, Chile, and Argentina are located [8,9]. This lineage classification is under review since information reported in the U.S. shows that both lineages are present in areas of California [35] and Arizona [26]. Ticks with characteristics of *R. sanguineus* s.l. have been reported in South America [36]. In Argentina *R. sanguineus* s.l. has been found infected with *A. platys* and *Rickettsia massiliae* [37]. It will be important to generate SDMs for each lineage once this issue with the species taxonomy is resolved.

## 5 Conclusions

The distribution models obtained for *R. sanguineus* suggest that by 2050, in scenarios 4.5 and 8.5, areas of habitat suitability for the tick will increase in the western U.S., Venezuela, Brazil, Uruguay, and Bolivia, and decrease in the Midwest and south of the US, Guyana, Peru, Bolivia, and Argentina. For 2070 the area of habitat suitability is predicted to increase regions of the western US, Brazil and Bolivia, and concomitantly decrease in the southern US, northern Brazil, Paraguay, and central Argentina. An increase in habitat suitability in tropical and subtropical regions of the Americas is predicted under the moderate emissions scenario 4.5 for both 2050 and 2070. Habitat suitability in the moist broadleaf forests and desert should remain constant; however, there will be losses in habitat in the FGS. In 2050 under the 8.5 high emissions scenario, habitat in TSCF and TGSS will remain constant; however, by 2070 there will be a loss of habitat suitability in TSMBF and an increase in habitat suitability in tropical and subtropical dry broadleaf forests. Our results may be useful in the design and implementation of effective surveillance programs for the control of the *R. sanguineus* tick and in establishing guidance to the public about the prevention of human exposure to this tick and its emerging diseases in the Americas.

## Supplementary Material

Supplementary Materials

## Figures and Tables

**Table 1:** Bioclimatic variables used for the construction of the ecological niche modelling for *Rhipicephalus sanguineus* and Variance inflation factor (VIF) [18] (https://www.worldclim.org accessed on 9 September 2022).

Bioclimatic Variables	Code	VIF
Mean Diurnal Range	BIO02	1.962970
Isothermality	BIO03	2.581188
Mean Temperature of Warmest Quarter	BIO10	1.191631
Precipitation of Wettest Month	BIO13	4.735302
Precipitation of Driest Month	BIO14	3.929649
Precipitation Seasonality	BIO15	3.774791
Precipitation of Warmest Quarter	BIO18	3.257963
Precipitation of Coldest Quarter	BIO19	2.930041

**Table 2:** Model calibration using Kuenm showing partial ROC, Omission %, and Delta AICc.

Pathway	Model	Partial ROC	Omision 5%	Delta AICc
2050_SSP2-4.5	M_0.4_F_1_Set_2	1.3367	0.0454	0.0000
2050_SSP5-8.5	M_0.9_F_1_Set_2	1.3856	0.0303	0.0000
2070_SSP2-4.5	M_0.4_F_1_Set_1	1.2829	0.0909	0.0000
2070_SSP5-8.5	M_0.4_F_1_Set_1	1.3637	0.0454	0.0000

**Table 3:** Percent change of geographic distribution for *Rhipicephalus sanguineus* between the model based on current environmental conditions and future projected climate change models for the years 2050 and 2070.

Observed	CanESM5		CanESM5	
	2050 SSP2-4.5	2050 SSP5-8.5	2070 SSP2-4.5	2070 SSP5-8.5
Increase	5.3	5.35	5.37	5.33
Persistence Presence	30.1	30.2	30.43	29.5
Persistence Absence	58.9	58.94	58.92	58.97
Reduction	5.6	5.51	5.28	6.2

CanESM5: Canadian Earth System Model version 5.

## Data Availability

The data presented in this study are available in the Supplementary Materials.
