# Supplementary material for: Predicting the Impact of Climate Change on the Distribution of Rhipicephalus sanguineus in the Americas
Source: Sustainability. Author manuscript; Available in PMC 2026 Feb 16. (PMC7618726; doi:10.3390/su15054557)
Supplement: Supplementary Materials [file EMS212471-supplement-Supplementary_Materials.pdf]

**Supplementary Materials:** The following supporting information can be downloaded at: https://www.mdpi.com/article/10.3390/su15054557/s1, Figure S1. Principal component analysis.
